# Androgenic Steroid Hormones and Endurance Exercise in Athletic Women

**DOI:** 10.3390/endocrines5030018

**Published:** 2024-07-02

**Authors:** Anthony C. Hackney, Raul Cosme Ramos Prado, Eimear Dolan

**Affiliations:** 1 Department of Exercise & Sport Science, University of North Carolina, Chapel Hill, NC 27599, USA; 2 School of Physical Education and Sport, University of São Paulo, São Paulo 05508-030, Brazil; 3 Applied Physiology and Nutrition Research Group-Center of Lifestyle Medicine, Faculdade de Medicina FMUSP, University of São Paulo, São Paulo 05508-030, Brazil

**Keywords:** anabolism, females, endocrine, adrenal, gonads, physical activity

## Abstract

This study investigated the impact of intensive endurance exercise on circulating androgenic steroid hormones in women. Fifteen normally menstruating athletic women participated. They completed intensive endurance exercise (treadmill running) until volitional fatigue in their follicular phase, with blood samples collected at pre-exercise, volitional fatigue, 90 min and 24 h into recovery. The steroid hormones (total, free testosterone, dehydroepiandrosterone [DHEA], and DHEA-sulfate [DHEA-S], cortisol) were analyzed in blood sera. Non-parametric statistics were used to assess changes across exercise and recovery. At volitional fatigue, all hormones, except free testosterone, were significantly (*p* < 0.05) increased compared to pre-exercise levels. Most hormones remained elevated through 90 min of recovery, with DHEA, DHEA-S, and total testosterone changes being significant (*p* < 0.05). At 24 h of recovery, hormonal levels were reduced; specifically, DHEA, DHEA-S, and total testosterone compared to baseline (*p* < 0.01 to 0.06). Increases in cortisol levels at volitional fatigue and 90 min of recovery were correlated with reductions in total testosterone, DHEA, and DHEA-S observed at 24 h of recovery (*rho* > −0.62, *p* < 0.05). In conclusion, in menstruating women performing intensive endurance exercise during their follicular phase, their androgenic steroid hormones remain elevated during early recovery but are suppressed at 24 h of recovery. The latter finding indicates that establishing a resting endocrine equilibrium requires a longer recovery period than 24 h.

## 1. Introduction

Circulating testosterone, a potent androgenic hormone, is typically 10–20 times lower in women than in men; however, the physiological relevance of this hormone in both sexes is well-established [1]. For men, the main source of circulating androgenic hormones is the gonads (testicles). In contrast, women derive circulating androgenic steroid hormones from both the adrenal gland and their ovaries (gonad). Specifically, dehydroepiandrosterone (DHEA), DHEA-sulfate (DHEA-S), and androstenedione from the adrenal glands [2], while testosterone is produced in roughly equal proportions by both the adrenal glands and the ovaries (~50:50; [2]) of women.

Evidence indicates there can be a suppression of androgenic steroid hormones following intensive exercise in men; the effect lasting from a few hours to several days [3-5]. One proposed mechanism for this hormone suppression is the exercise-induced elevation in cortisol causing direct gonadal steroidogenesis inhibition and/or disruption of the hypothalamic–pituitary–gonadal (HPG) axis regulation of gonadal function [6,7].

The influence of intensive exercise on circulating androgenic steroid hormones in women is far less studied or as well understood as compared to men. As such, existing evidence on exercise effects often lacks consensus [8-13]. This lack of consensus in the few existing studies may be due to several key factors: (1) the lack of control for the menstrual cycle phase or the hormonal status (e.g., oral contraception use) of women participants, and/or (2) aspects of sex-related physiological differences such as body fat which can affect circulating steroid hormones via peripheral aromatization [13,14].

Because of the limited amount of research on women on this topic, and the overall physiological criticality of androgenic hormones to a woman’s health and well-being [15], our group examined whether intensive exercise would have the same effect on androgenic steroid hormones in women as in men. As such, the purpose of our study was to examine the influence of intensive endurance exercise on a comprehensive androgenic steroid hormone profile in well-trained women athletes. We chose to investigate endurance exercise since prior research demonstrated that such exercise could invoke robust changes in androgenic hormones [16], as well as due to the popularity of endurance activities in sports women [17].

## 2. Materials and Methods

### 2.1. Study Subjects

Fifteen young healthy women, between 18 to 26 years old, completed this study. The participants were aerobically trained endurance runners or triathletes, having trained for a minimum of 3 years, 5 days per week, for at least 60 min a day. Based on performance characteristics, the participants were rated at the Tier 3 level according to the McKay et al. classification system [18]. They were free of any known endocrine disorders, health problems, or musculoskeletal injuries based on a medical screening. In this report, we use the term women as an indicator of biological sex (female) and in the context of a cis-gender representation. All participants gave their informed consent for inclusion before they became involved in the study. The study was conducted in accordance with the Declaration of Helsinki, and the protocol was approved by the Ethics Committee of the University of North Carolina (Institutional Review Board-Project identification number—10-2109-PEES).

### 2.2. Study Design

Participants came to our laboratory and completed three study sessions. These sessions involved (1) a maximal oxygen uptake (VO_2max_) and body composition assessment, (2) an intensive treadmill running session to volitional fatigue, and (3) a recovery session (24 h post-exercise). All laboratory sessions were performed during the early follicular phase of their menstrual cycle (within 3–4 days of menses ending) and occurred in two consecutive menstrual cycles, with session one performed in the first cycle and sessions two and three in the second cycle. The follicular phase of the menstrual cycle was chosen due to the stable hormonal profile status during this period [19]. To ensure participants were naturally menstruating, before the study began, their menstrual cycle status was monitored for three months using daily oral basal body temperature records (self-reports) to confirm ovulation occurred [20] (menstrual cycle length = 29.3 ± 2.0 days, ovulation = 13.0 ± 3.1 days of cycle). This research was conducted with the approval of the institutional ethical committee, and all participants gave written informed consent. Subject characteristics appear in Table 1.

### 2.3. Session One—Maximal Oxygen Uptake/Body Composition

At this session, participants completed their informed consent, a training history questionnaire, and a medical history questionnaire (MHQ), and a physical screening was performed. The physical screenings involved a resting 12-lead echocardiogram, blood pressure measurements, and basic assessments of pulmonary, circulatory, orthopedic function, and a review of their MHQ. Height in centimeters (cm) and body mass in kilograms (kg) were recorded. Skinfolds were assessed at three sites (triceps, supra-iliac, and femoral) using Harpenden skinfold calipers following the protocol for the Jackson–Pollock skinfold regression equation protocol to estimate the percentage of body fat [21].

A modified Åstrand treadmill test (as reported by Pollock and Wilmore [22]), using a Quinton Q65 series 90 treadmill (Quinton Instrument Company, Vista, CA, USA) was used to determine VO_2max_. The starting speed for the treadmill was determined from information provided on the training history questionnaire. The grade of the treadmill was kept constant and fixed at 1.5%, while the speed was increased incrementally every 3 min. Respiratory gases (oxygen uptake [VO_2_], carbon dioxide production [VCO_2_]) were measured using a TrueMax 2400 analyzer system (Parvo Medics, Murray, UT, USA), and data were collected every 15 s during the testing. Heart rate (HR; in beats per minute; bpm) was recorded every minute of exercise, and the rating of perceived exertion (RPE; scale 6–20) was recorded at the end of every three minutes [23]. The VO_2max_ test was terminated upon volitional fatigue. Participants were determined to have reached VO_2max_ based upon achieving previously published criteria (respiratory exchange ratio > 1.10, heart rate maximal within 5% [age predicted max], RPE > 18, and a plateau or reduction in VO_2_ with increasing workload [22,24].

### 2.4. Session Two—Prolonged Exercise

Before arrival at the laboratory, participants were asked to (1) not participate in any strenuous activity or ingest any alcohol for the prior 24 h, (2) not consume any caffeine for 12 h prior, and (3) report in a 3 h fasted state. For the three days before this exercise session, they were asked to eat a diet in which at least 60% of the caloric intake was from carbohydrates. A 3-day dietary recall was used to ensure the participants followed these guidelines.

The prolonged exercise session took place at a time (12:00 pm to 3:00 pm) chosen to ensure cortisol concentrations were relatively stable (i.e., avoiding large circadian rhythm fluctuation; [25]). A 24-gauge catheter (BD Vacutainer Systems, Washington, DC, USA) was inserted in an antecubital vein for blood sampling. Participants then rested in a supine position for 30 min; then, a baseline pre-exercise blood sample (3 mL) was taken. They were next fitted with a Polar heart rate monitor (Polar, Helsinki, Finland) and began a 10 min warm-up protocol. The warm-up protocol consisted of cycling on a Monark cycle ergometer (Monark 814 Ergomedic, Stockholm, Sweden) for 3 min at 60 revolutions per minute (rpm) with 1 kp resistance, followed by 5 min of stretching. It concluded with 2 min of light walking on the treadmill.

Following the warm-up protocol, participants ran on the treadmill at a running speed to elicit their pre-determined ventilatory threshold (VT; ±5%; *N.B.*, representing VT one [24]) until they reached the point of volitional fatigue—the intent here was to approximate the intensity a subject would experience if running a half-marathon race [26]. The ventilatory threshold for each subject was determined from their VO_2max_ test [24]. Respiratory gases, HR, and RPE data were collected at 5, 30, and 60 min during the exercise to monitor the intensity (i.e., adjustments in running speed were performed as needed). After 60 min, these measures were taken every 15 min (not reported herein) and again when the subject reached volitional fatigue. Blood lactate concentration was measured only at the point of volitional fatigue and used to further confirm that the participants were performing intense exercise. Post-exercise blood samples (3 mL) were taken at volitional fatigue and 90 min of recovery after the participants rested in a supine position for 90 min.

Participants were verbally encouraged as they approached the point of fatigue as well as throughout the exercise. During the exercise and recovery period, the participants were allowed to drink water *ad libitum*.

### 2.5. Session Three—24 h Recovery Assessment

Participants returned to the laboratory approximately 23.5 h from their point of volitional fatigue, then rested in a supine position for 30 min, after which a final blood sample (3 mL) was taken (24 h recovery). They were instructed not to participate in any strenuous activity, not to ingest any alcohol or caffeine, and to eat and drink ad libitum for 24 h before reporting to our laboratory.

### 2.6. Biochemical Analysis

Blood samples were collected into sterile vacutainer tubes (BD Vacutainer Systems, USA) and placed on ice immediately. A small sample of whole blood was transferred into EDTA-treated tubes (BD Vacutainer Systems, USA) to be analyzed for hematocrit and hemoglobin. Plasma volume shifts were calculated from these measurements to account for blood hemoconcentration [27]. Hematocrit was determined in triplicate using the microcapillary tube method. Hemoglobin was assessed via colorimetric determined using a cynamethoglobin reaction quantified on a spectrophotometer (Milton Roy, Sacramento, CA, USA). After clotting, the remaining blood samples were later centrifuged at 3000× *g* at 4 °C to separate sera which were stored in cryo-freeze tubes at −80 °C until analysis. Blood serum was analyzed using single antibody solid phase radioimmunoassay procedures (triplicate determination) with ^125^I, using commercially available kits for the determination of cortisol, total testosterone, free testosterone, DHEA, and DHEA-S (Siemens-DPC Inc., Washington, DC, USA).

Blood lactate was assessed in duplicate on select blood samples using dry chemistry colorimetric procedures involving a Vitros DT-60 analytical unit (Johnson & Johnson, Santa Clara, CA, USA).

### 2.7. Statistical Analysis

Statistical analyses were conducted using the SPSS software program (v23.0, Chicago, IL, USA). Descriptive statistics were determined for participant characteristics, including age, height, body mass, percent body fat, and VO_2max_ (see Table 1). Significant changes in hormone concentrations across exercise and recovery time were assessed with the Friedman repeated measures analysis of variance (ANOVA) with pairwise comparison post hoc tests (Bonferroni correction) being applied where appropriate. Spearman *rho* correlation coefficients were conducted to examine relationships between hormone concentrations. The level of statistical significance was set at *p* ≤ 0.05. Non-parametric analytical approaches were used due to the non-normal distribution of hormonal data as previously recommended [28].

## 3. Results

### 3.1. Endurance Exercise Responses

The mean time to volitional fatigue was 91.2 ± 9.1 min (± SD) with an average running speed of 14.3 ± 0.7 km/h. Table 2 displays the HR, percentage (%) VT, lactate, and RPE during this exercise.

### 3.2. Hormonal Responses

Hormonal responses are reported in Table 3. There was a significant increase in all hormone concentrations (except free testosterone) at volitional fatigue compared to pre-exercise. Similarly, all hormones (except total testosterone) remained elevated at 90 min of recovery compared to pre-exercise (*N.B.*, DHEA significance level was *p* < 0.06). There was a significant reduction in all hormone concentrations at 24 h of recovery compared to pre-exercise (*N.B.*, total testosterone significance level was *p* < 0.06).

### 3.3. Plasma Volume

There was a significant, but transient, reduction of −9.6% (±4.1) plasma volume from pre-exercise to volitional fatigue (*p* < 0.01). This magnitude of change in plasma volume, in comparison to the alterations in hormonal concentrations, suggests that hemoconcentration was not the primary factor influencing the hormonal concentration increases during exercise [28]. At 90 min of recovery, there was a slight increase (+2.3 ± 3.7%; *p* > 0.05) in plasma volume, leading to a minor degree of hemodilution. Plasma volume was again reduced (−2.7% ± 4.1) at 24 h of recovery compared to pre-exercise, but this was not statistically significant (*p* > 0.05).

### 3.4. Correlation Analysis

Spearman correlation (*rho*) revealed a relationship between the increases in cortisol levels (both volitional fatigue and at 90 min of recovery) with the reductions observed in total testosterone, DHEA, and DHEA-S at 24 h of recovery (*rho* ranged from −0.63 to −0.69, *p* < 0.02 to *p* < 0.01).

## 4. Discussion

The objective of this study was to examine the response of select androgenic steroid hormones to intensive endurance exercise in sportswomen. Moreover, our focus was on determining whether these hormones exhibit similar patterns of behavior in women as those previously reported in men when performing comparable exercise. We addressed this research question because of a limited amount of investigative work available on this aspect of androgenic endocrinology concerning women engaging in sports.

Our findings indicate women have increased concentrations of androgenic hormones at volitional fatigue as well as 90 min in recovery after a prolonged, intense endurance exercise. Similar results for some of the hormones assessed herein were observed in women/female participants in non-competitive (laboratory) endurance exercise [29-32] as well as in competitive exercise activities (non-laboratory) [30,33-35]. These hormonal increases can be attributed, to some extent, to a degree of hemoconcentration (i.e., reductions in plasma volume), along with a combination of increased hormonal production and reduced metabolic clearance [33,36,37]. In men, comparable endurance exercise demonstrated an acute increase in total testosterone within 30 min of the exercise bout ending, and with subsequent reductions in free and total testosterone by 24 h after the exercise [4]. That is, analogous to the present data.

As just noted, our 24 h recovery findings are similar to prior results in men [3,4]. Nevertheless, in looking at male versus female outcomes, it is important to recognize that the magnitude of some of the absolute hormonal concentrations observed in these studies varies greatly from those of the present data due to sex-related variations in circulating hormone concentrations [1,9] (e.g., we speculate the lack of significant reductions in free testosterone due to the already low levels of the hormone, and hence, the occurrence of a statistical “basement effect”; [38]). From our perspective, our finding of reduced hormone levels at 24 h into the recovery is our most critical finding (see later discussion). It is unclear if the observed reductions are a compensatory feedback regulatory suppression following substantial hormonal increases in response to the exercise bout [37], or a regulatory axis disruption [37,39]. Relative to this last point, Cumming et al. [6] reported that cortisol elevations (in men) can lead to a suppression of androgenic hormones such as testosterone (via direct steroidogenesis inhibition of androgen production), and research also supports that the HPG axis regulation can be disrupted by high cortisol levels [7]. In the case of women, however, considering the significant contribution of both gonadal and adrenal glands to androgen hormone production, precursor availability may also play a role in our findings. That is, at the adrenal gland, pregnenolone serves as a precursor for cortisol as well as the other androgenic hormones produced by this gland (diverging biochemical pathways). Consequently, the increased production of cortisol could have led to a reduction in other adrenal androgenic hormone production, as pregnenolone may have been diverted toward cortisol biosynthesis [40]. This premise is supported by the significant negative correlation between the elevated cortisol levels we found and the observed reductions in androgenic hormones. We acknowledge, nonetheless, that this point is speculative on our part and additional work is necessary to support or refute this supposition.

We further examined the relationship between cortisol and the androgenic hormones in an exploratory post hoc statistical analysis to determine if the reductions at 24 h were solely due to elevations in cortisol or perhaps the resultant of exercise performance (i.e., the varying time to volitional fatigue). As such, we recalculated the Spearman *rho* coefficients using a partial correlation analysis approach (with time to volitional fatigue as the covariate). By controlling for time to fatigue (performance) in this fashion, we assumed that if it were performance driving the relationships, then the correlation coefficients would become reduced and perhaps non-significant. This new analysis did not reveal such an effect to any great degree; hence, we surmise that cortisol levels/changes seem to be more influential on the observed relationships to the androgenic hormones.

From a practical perspective, each of the hormones we examined plays a central role in mediating the physiological changes that lead to exercise training adaptations as well as the overall health/well-being in women [15]. Hence, the hormonal reductions at the 24 h point could potentially impact the time course of adaptative changes. In other words, our findings suggest the endocrine system is not completely recovered in a 24 h window of time in sportswomen following such intensive exercise. As such, after engaging in such exercise activities, adequate rest and recovery are crucial to the athlete. Specifically, how much rest is needed is a question that should be addressed fully in future research.

We acknowledge there are limitations within the current study. For example, a more robust hormonal profile such as the inclusion of androstenedione could have provided more physiological insight. Additionally, an examination of hormonal responses across the menstrual cycle phases (i.e., ovulation and luteal phases) would have perhaps revealed more information on the complexities of the female reproductive endocrine system and its interactions with exercise. Regrettably, both financial and time constraints did not allow us to address these points. Finally, the current data findings are delimited in their application, as it is uncertain if women of a lesser or greater level of physical fitness (e.g., elite athletes) would respond similarly or not.

To conclude, the responses of androgenic hormones to intensive endurance exercise in women closely resemble what previous research has observed in men/males. The findings demonstrate that in menstruating women, during their follicular phase, androgenic steroid hormones remain elevated in the early recovery period following the termination of such activity, but are significantly reduced for 24 h afterward. We surmise this later outcome indicates the endocrine system is not fully recovered from exercise as used herein, and longer than 24 h is needed to reestablish hormonal equilibrium.

## Figures and Tables

**Table 1.** Characteristics of the subjects. Abbreviations: VO_2max_ = maximal oxygen uptake, VT = ventilatory threshold.

Measurement (Unit)	Mean	Standard Deviation
Age (y)	26.5	2.4
Height (cm)	169.7	6.0
Mass (kg)	60.8	3.9
Body Fat (%)	18.4	3.7
Training (y)	7.7	2.1
VO_2max_ (mL/kg/min)	59.2	2.7
VO_2_ at VT (% of VO_2max_)	76.5	4.2

**Table 2.** Select results (mean ± SD) for the prolonged run to volitional fatigue (* indicates significant differences from minute 5 values, *p* ≤ 0.05). HR = heart rate, VT = ventilatory threshold, RPE = rating of perceived exertion, a.u. = arbitrary units.

Measure (Unit)	Exercise Time			
	5 min	30 min	60 min	Volitional Fatigue
HR (bpm)	162.2 ± 5.8	174.6 ± 6.3	175.8 ± 10.0	179.3± 7.1 *
VT (%)	92.7 ± 5.6	96.6 ± 5.1	97.1 ± 4.9	99.8 ± 6.7 *
Lactate (mM/L)	--	--	--	5.9 ± 0.6
RPE (a.u.)	11.3 ± 1.9	13.9 ± 2.1	15.0 ± 1.9	16.8 ± 2.5 *

**Table 3.** Hormone concentrations before and after the intensive endurance exercise in women runners. Values are mean (± SD). Probability (*p*) levels are below mean values and indicate significance from the pre-exercise (resting) values. NS = non-significant, DHEA = dehydroepiandrosterone, DHEA-S = dehydroepiandrosterone-sulfate.

Hormone (Unit)	Measurement Time			
	Pre-Exercise (Resting)	Volitional Fatigue	90-min Recovery	24-h Recovery
Cortisol (μg/dL)	19.5 ± 6.0	33.7 ± 5.1 <0.01	28.9 ± 6.1 <0.01	16.7 ± 5.9 NS
Total Testosterone (ng/dL)	16.7 ± 7.1	29.9 ± 8.8 <0.01	18.9 ± 4.4 NS	12.5 ± 4.0 <0.06
Free Testosterone (pg/mL)	0.9 ± 0.7	1.7 ± 1.9 0.08	1.6 ± 1.0 <0.03	0.6 ± 0.9 NS
DHEA (μg/dL)	210.5 ± 44.3	279.0 ± 66.3 <0.01	255.0 ± 75.3 <0.06	169.1 ± 55.9 0.03
DHEA-S (μg/dL)	123.7 ± 25.1	191.1 ± 65.1 <0.01	137.6 ± 69.9 0.04	88.9 ± 33.0 <0.01

## Data Availability

Contact the senior author about data availability.
